# Genomic Basis of Zoonotic Transmission and Antifungal Resistance in *Microsporum canis*

**DOI:** 10.3390/jof12060429

**Published:** 2026-06-11

**Authors:** Zebin Du, Yuling Zhang, Xinting Meng, Zexun Lv, Yang Wang, Congming Wu

**Affiliations:** 1 College of Veterinary Medicine, China Agricultural University, Beijing 100193, China; 2 Department of Pharmacology and Toxicology, College of Veterinary Medicine, China Agricultural University, Beijing 100193, China

**Keywords:** *Microsporum canis*, dermatophytosis, zoonotic transmission, antifungal resistance, whole-genome sequencing, comparative genomics, ABC transporter, *CDR1*

## Abstract

*Microsporum canis* is a globally prevalent zoonotic dermatophyte and the major causative agent of dermatophytosis in both pets and humans. The widespread clinical use of antifungal drugs has led to the frequent emergence of decreased susceptibility, while its molecular features and the genomic basis of cross-host transmission remain incompletely elucidated. In this study, 38 clinical *M. canis* isolates were collected from dogs and cats in Beijing (2025). We determined the minimum inhibitory concentrations (MICs) of six common antifungal agents via microbroth dilution, and performed whole-genome sequencing and comparative genomic analysis. All isolates showed high clonal homogeneity, with ANI >99.9% to the reference. A local human-derived strain was nested within the pet-derived clade, supporting zoonotic cross-host transmission. Terbinafine exhibited the highest activity, while itraconazole, voriconazole, posaconazole, griseofulvin, and ciclopirox olamine showed higher MICs; 11 isolates showed a multidrug high-MIC phenotype. Notably, copy number variation in the ABC transporter gene *CDR1* was positively correlated with MICs of multiple antifungal agents (p<0.05). This study provides a genomic basis for optimized antifungal therapy, resistance surveillance and transmission control of zoonotic *M. canis*.

## 1. Introduction

Dermatophytosis is one of the most prevalent superficial fungal infections worldwide. *Microsporum canis*, a prototypical zoonotic dermatophyte, is the major causative agent of dermatophytosis in dogs and cats. Epidemiological studies consistently report isolation rates exceeding 90% in feline cases and over 60% in canine cases across different regions [1,2,3,4]. Moreover, *M. canis* is a leading pathogen of human dermatophytosis, particularly tinea capitis in children, and its prevalence has been increasing in several regions worldwide [5,6,7]. Close contact between companion animals and humans facilitates cross-host transmission, making this fungus a key driver of intrafamilial zoonotic infection and a sustained public health concern [8,9,10].

To date, numerous studies have characterized the virulence factors and antifungal susceptibility profiles of *M. canis* [11,12,13,14,15]. Clinically, antifungal agents including ciclopirox olamine, griseofulvin, azoles, and allylamines are widely used. However, their extensive use has led to the frequent emergence of elevated MICs and reduced susceptibility, particularly to first-line drugs such as terbinafine and itraconazole [13,16,17], contributing to treatment failure in up to 40% of patients [11]. At the mechanistic level, virulence factors, including secreted proteolytic enzymes, lipases, oxidative stress tolerance-related molecules, and efflux pump proteins [14], have been implicated in host colonization, invasion, and immune evasion [12].

Despite these advances, several key gaps remain. Most antifungal susceptibility data and molecular mechanistic studies focus on human-derived strains, whereas systematic data from companion animal-derived epidemic strains remain scarce [8,13,18]. Comparative genomic studies of *M. canis* are limited, and the population genetic structure as well as the genomic distribution of core virulence factors are not well characterized [15,19]. Importantly, although companion animals are recognized as major reservoirs for the zoonotic transmission of *M. canis* [3], genome-based phylogenetic evidence linking animal-derived and human-derived strains, and clarifying the molecular basis of cross-host transmission, is still lacking [9].

Therefore, the primary objective of this study was to perform an integrated phenotypic and genomic analysis of 38 clinical *M. canis* isolates from dogs and cats at two Beijing veterinary hospitals in 2025. Specifically, we aimed to: (i) determine the susceptibility profiles to six common clinical antifungals using the latest CLSI M38 (3rd Edition) broth microdilution method [20]; (ii) conduct whole-genome sequencing and comparative genomic analyses to resolve the population structure and characterize virulence- and resistance-related genes; and (iii) assess zoonotic transmission potential via phylogenetic comparisons with human-derived reference strains.

## 2. Materials and Methods

### 2.1. Strain Source and Identification

A total of 38 *M. canis* isolates were collected from companion animals diagnosed with dermatophytosis at two veterinary hospitals in Beijing from April to October 2025. The host origin of these isolates included 16 from dogs (*Canis lupus familiaris*) and 22 from cats (*Felis catus*). All clinical samples were obtained from the junction of diseased and healthy skin (hairs and scales) of animals preliminarily diagnosed via Wood’s lamp examination and/or direct microscopic observation by clinical veterinarians. As the primary focus of this study was on the in vitro antifungal susceptibility and genomic characteristics of the pathogens rather than clinical epidemiology, detailed clinical signs (e.g., specific lesion types, extent of alopecia, or other systemic signs) and exact lesion locations were not systematically recorded. Prior to sampling, the affected area was shaved and disinfected with 75% ethanol, and hair with intact follicles showing characteristic yellow-green fluorescence was aseptically collected. All sample collection procedures were approved by the Teaching and Research Department of China Agricultural University Veterinary Teaching Hospital (approval No. 202501041709000223875, approval date: 4 January 2025). All samples were collected from client-owned animals with written informed consent from the pet owners, and all procedures were performed in strict accordance with institutional and national guidelines for the care and use of companion animals.

Samples were inoculated on Sabouraud dextrose agar (SDA) medium and incubated at 28 °C for 3–7 days. SDA was utilized instead of Mycosel agar based on two main considerations: first, SDA is a universally recognized and widely applied standard medium for the cultivation and maintenance of dermatophytes [21,22]; second, considering the large sample size in the initial screening phase, SDA offered a more cost-effective alternative to the relatively expensive Mycosel agar. Potential contamination by non-dermatophyte fungi was effectively managed through strict aseptic techniques and subsequent molecular identification. Preliminary identification was performed based on colony morphological characteristics and microscopic observation of spiny, thick-walled fusiform macroconidia (≥6 cells) according to the standard criteria described in the *Atlas of Clinical Fungi* [21]. Single colonies were purified by three successive passages on SDA medium to obtain pure cultures. Genomic DNA was extracted from each purified strain, and the internal transcribed spacer (ITS) region was amplified by PCR using the universal primers ITS1 (5′-TCCGTAGGTGAACCTGCGG-3′) and ITS4 (5′-TCCTCCGCTTATTGATATGC-3′) [23]. PCR amplification was performed in a 25 μL reaction system containing 12.5 μL of 2 × Taq PCR MasterMix (Tiangen Biotech, Beijing, China), 1 μL each of 10 μmol/L forward and reverse primers, 2 μL of template genomic DNA (20–50 ng/μL), and 8.5 μL of nuclease-free water. The amplification protocol was as follows: initial denaturation at 95 °C for 5 min; 35 cycles of denaturation at 94 °C for 30 s, annealing at 55 °C for 30 s, and extension at 72 °C for 1 min; and a final extension at 72 °C for 10 min.

PCR products were verified by 1.0% agarose gel electrophoresis to confirm a single target band of approximately 550 bp. Qualified products were subjected to Sanger sequencing by Beijing Novogene Technology Co., Ltd. (Beijing, China). The obtained sequences were aligned against the NCBI Nucleotide database using BLASTn v2.14.0+ [24], with a sequence similarity of ≥98% as the final species confirmation criterion.

### 2.2. Antifungal Drug Susceptibility Test

The in vitro antifungal susceptibility of 38 *M. canis* isolates to six antifungal agents (ciclopirox olamine, griseofulvin, posaconazole, terbinafine, voriconazole, and itraconazole) was determined via the broth microdilution method, in strict accordance with the CLSI M38 (third edition) standard [20]. All tested antifungal agents were obtained as pure standard powders from Aladdin Biochemical Technology Co., Ltd. (Shanghai, China).

All isolates and the quality control strain *Trichophyton mentagrophytes* ATCC MYA-4439 were activated on SDA medium at 28 °C for 5 days. Subsequently, 1 mL of sterile 0.85% saline was added to the culture surface and allowed to soak for 1–2 min. The surface was gently scraped with a sterile inoculation loop to dislodge hyphae and spores. The resulting suspension was collected and left to stand at room temperature for 20 min to allow larger hyphal fragments to settle at the bottom. The upper homogeneous liquid was then filtered through sterile No. 40 filter paper to remove residual debris. A 10 μL aliquot of the filtrate was used to count the spores using a hemocytometer under a microscope. The spore concentration was adjusted using MOPS-buffered Roswell Park Memorial Institute-1640 (RPMI-1640) medium (pH 7.0) to obtain a final working inoculum of 2–6 $\times \;10^{3}$ CFU/mL. To verify the accuracy of the inoculum concentration, 0.01 mL of the prepared suspension was evenly spread onto an SDA plate, incubated at 30 °C for 3 days, and the resulting colonies were counted.

Drug stock solutions were prepared with dimethyl sulfoxide (DMSO) and serially diluted with RPMI-1640 medium, with a final DMSO concentration ≤1% (*v*/*v*) to avoid solvent interference. The final concentration gradients were set as follows: 0.004–16 μg/mL for ciclopirox olamine, posaconazole, voriconazole, and itraconazole; 0.008–32 μg/mL for griseofulvin; 0.001–4 μg/mL for terbinafine. For the microdilution assay, 100 μL of the standardized spore suspension was added to wells 1–11 of a 96-well microtiter plate containing the serially diluted antifungal drugs. Each isolate was tested in duplicate, with positive growth and blank medium controls included on each plate. Plates were incubated at 30 °C for 96 h. The minimum inhibitory concentration (MIC) was defined as the lowest drug concentration achieving ≥80% growth inhibition relative to the positive control, consistent with the endpoint criteria validated for dermatophytes under the CLSI M38 (third edition) framework.

The high-MIC phenotype (non-wild type, NWT), defined as decreased in vitro antifungal susceptibility, was designated when an isolate’s MIC exceeded the upper limit of wild-type (UL-WT) threshold for the corresponding antifungal agent. UL-WT thresholds were determined with reference to a 27-year large-scale MIC study of 348 *M. canis* isolates from mainland China, the largest epidemiological dataset of this pathogen in China to date [13]. Thresholds were set as follows: terbinafine ≥ 0.125 μg/mL, itraconazole ≥ 0.25 μg/mL, voriconazole ≥ 0.125 μg/mL, posaconazole ≥ 0.25 μg/mL, and griseofulvin ≥ 2 μg/mL. For ciclopirox olamine, which was not included in the aforementioned study, the threshold was set as ≥2 μg/mL with reference to Chinese veterinary clinical guidelines for dermatophytosis and published dermatophyte susceptibility studies.

A multidrug high-MIC phenotype was defined as an isolate exhibiting a high-MIC phenotype against two or more classes of antifungal agents with distinct mechanisms of action. The tested agents were classified into four categories based on their mechanism of action: (1) allylamines (terbinafine, squalene epoxidase inhibitor); (2) triazoles (itraconazole, voriconazole, posaconazole, lanosterol 14α-demethylase inhibitor); (3) hydroxypyridones (ciclopirox olamine, metal ion chelator); (4) griseofulvins (griseofulvin, tubulin inhibitor) [25].

*Trichophyton mentagrophytes* ATCC MYA-4439 was tested in parallel with each batch of test isolates. All measured MIC values of the quality control strain fell within the CLSI-specified acceptable ranges, confirming the reliability of all test results.

### 2.3. Whole-Genome Sequencing, Assembly and Quality Assessment

Genomic DNA was extracted from pure *M. canis* cultures using a modified cetyltrimethylammonium bromide (CTAB) method as previously described [26]. The integrity and purity (A260/A280 = 1.8–2.0) of the extracted DNA were verified via 1.0% agarose gel electrophoresis and NanoDrop 2000 detection. Qualified DNA was fragmented by sonication to construct 350 bp insert paired-end libraries, which were validated using an Agilent 5400 system and qPCR, then sequenced on the Illumina NovaSeq 6000 platform with 150 bp paired-end reads.

Raw reads were quality-filtered using fastp v0.23.4 [27] with the following criteria: removal of adapter-contaminated reads, reads with >10% ambiguous bases (N), and reads with >50% low-quality bases (Phred score < 5). Briefly, a total of 141 Gb of raw data was generated, with an average of 3.71 Gb clean data per isolate (mean Q20 = 99.42%, Q30 = 97.35%, GC content = 47.45%, average sequencing depth = 163×), meeting the requirements for high-quality fungal genome assembly. Detailed sequencing quality metrics and genome accession numbers for all isolates are presented in Table 1.

De novo genome assembly was performed using SPAdes v3.15.5 [28]. Assembly metrics were assessed using QUAST v5.2.0 [29], genome completeness was evaluated via BUSCO v5.4.7 [30] with the fungi_odb10 dataset, and host contamination was filtered via Blastn v2.14.0+ alignment [24]. Species identity was further validated via average nucleotide identity (ANI) analysis against the *M. canis* ATCC 4439 reference genome (GCF_000151145.1) using FastANI v1.33 [31]. Two human-derived *M. canis* genomes (GCA_026259285.1 and GCA_051943485.1) were downloaded from the NCBI Assembly database, processed through the same analytical pipeline, and included in subsequent comparative genomic analyses.

### 2.4. Genome Annotation and Phylogenetic Analysis

Whole-genome annotation was performed using the Funannotate v1.8.16 pipeline [32]. Briefly, genomic repetitive sequences were masked using RepeatMasker, and protein-coding gene prediction was performed using Augustus v3.4.0 [33] based on the fungal universal model. Gene function annotation was completed by alignment against the Swiss-Prot (v2026.01), Pfam (v35.0), and EggNOG (v5.0) databases, and a standardized GFF3 format annotation file was generated through integration.

Phylogenetic analysis was constructed based on core single-nucleotide polymorphisms (cgSNPs). Using *M. canis* ATCC 4439 as the reference genome, alignment of all strain genome sequences was performed using Snippy v4.6.0 [34]. Core genome alignment sequences were extracted using snippy-core, and high-quality cgSNP sites were obtained using snp-sites v2.5.1 [35] after quality control filtering of invalid sites. A maximum likelihood (ML) phylogenetic tree was constructed using IQ-TREE v2.4.0 software [36], with the best-fit evolutionary model automatically selected by ModelFinder [37] and 1000 ultrafast bootstrap tests set to evaluate branch reliability. The iTOL v7 online tool [38] was used to integrate metadata including strain host source and drug susceptibility phenotype for visualization of the phylogenetic tree.

### 2.5. Mining of Virulence- and Drug Resistance-Related Genes

Virulence and antifungal resistance-related genes were identified via homologous alignment using BLASTp v2.14.0+ [24]. Predicted protein sequences of each isolate were aligned against two databases: (1) the Fungal Pathogen Virulence Factor Database (DFVF) [39] for virulence gene identification; (2) a custom curated UniProt-based database of experimentally validated fungal antifungal resistance-related proteins (detailed in Supplementary Table S1), covering ergosterol biosynthesis, cell wall biosynthesis, ABC/MFS efflux pumps, and stress response regulation.

BLASTp alignment thresholds were set as E-value $\leq 10^{-5}$, sequence identity > 40%, and alignment length > 200 amino acids, which are widely validated in fungal comparative genomics to minimize false positives while retaining accurate homologous matches [39]. High-quality alignments were screened using the Best-Hit strategy.

Virulence gene analysis focused on core elements including ABC/MFS transporters, secreted subtilisins (SUB), metalloproteases (MEP), ergosterol/cell wall biosynthesis-related genes, and heat shock proteins. Resistance-related gene analysis focused on core genes including efflux pumps (*CDR1*, *PDR5*), ergosterol biosynthesis targets (*ERG1*, *ERG11*), cell wall synthesis gene (*FKS1*), and stress response gene (*HSP90*). A copy number matrix of target genes for each isolate was constructed based on the filtered alignments.

### 2.6. Statistical Analysis

All data analysis and visualization were performed using R v4.3.1 software [40]. The chi-square test with continuity correction or Fisher’s exact test was used to compare differences in the proportion of high-MIC isolates between *Canis lupus familiaris*-derived and *Felis catus*-derived strains, with the test method selected according to the expected frequency of the 2 × 2 contingency table. Spearman rank correlation analysis was performed to assess the association between resistance gene copy number and log_2_-transformed MIC values, with the Spearman correlation coefficient (r) and 95% confidence interval (CI) reported for each gene–drug pair. Univariate linear regression was conducted to quantify the proportion of MIC variance explained by *CDR1* copy number variation (CNV), with the coefficient of determination ($R^{2}$) reported. The Kruskal–Wallis nonparametric test was used to compare differences in log_2_-transformed MIC values among groups with different *CDR1* copy numbers.

The pheatmap package v1.0.13 [41] was used to generate heatmaps of virulence and drug resistance gene distribution, with strain clustering analysis performed based on Euclidean distance and the Complete linkage clustering algorithm. The ggplot2 v4.0.3 package [42] was used to generate correlation bubble plots and box plots of drug susceptibility distribution.

## 3. Results

### 3.1. Epidemiological Characteristics, Strain Identification and Phylogenetic Analysis

**Figure 1:**
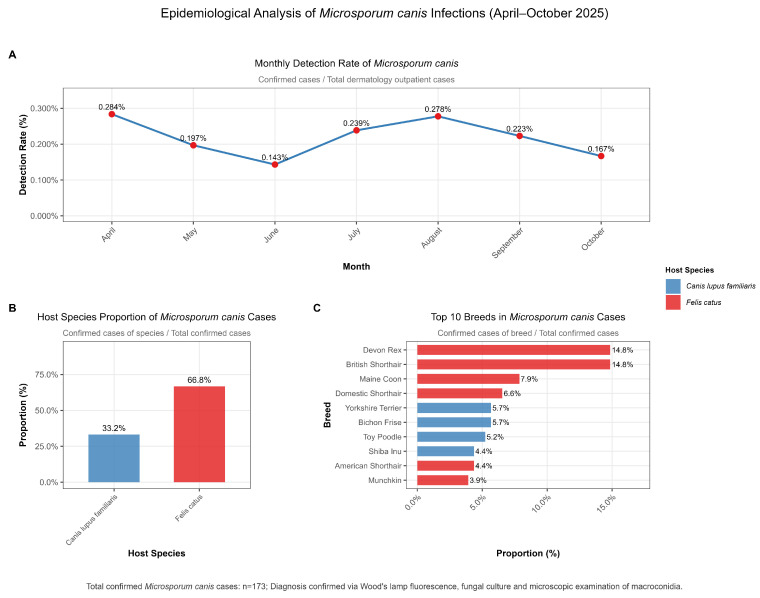
Epidemiological characteristics of *Microsporum canis* infections in Beijing, April to October 2025. (**A**) Monthly detection rate of *M. canis* in the monitored veterinary hospitals, calculated as the number of confirmed cases divided by total dermatology outpatient cases in the corresponding month. (**B**) Host species distribution of confirmed *M. canis* infection cases, showing the proportion of *Canis lupus familiaris* and *Felis catus* hosts. (**C**) Top 10 affected breeds of confirmed *M. canis* infection cases, with the proportion of each breed in total confirmed cases shown. Total confirmed *M. canis* cases: n = 173; diagnosis confirmed via Wood’s lamp fluorescence, fungal culture, and microscopic examination of macroconidia.

Between April and October 2025, a total of 79,696 dermatology outpatient cases were enrolled from the two monitored veterinary hospitals in Beijing, among which 173 cases were etiologically confirmed as *M. canis* infection (Figure 1). The monthly detection rate of *M. canis* fluctuated between 0.14% and 0.28%, with two distinct incidence peaks in April and August (Figure 1A). Host distribution analysis showed that *Felis catus* was the predominant infected host, accounting for 65.4% of total confirmed cases (n = 113), while *Canis lupus familiaris* accounted for 34.6% of cases (n = 60) (Figure 1B). Breed distribution analysis revealed that British Shorthair cats and Toy Poodles represented the highest proportion of confirmed cases within feline and canine populations, respectively (Figure 1C). From the 173 confirmed cases, 38 strains were successfully isolated and preserved for subsequent whole-genome sequencing (WGS) and in vitro antifungal susceptibility testing.

All 38 isolates were confirmed as *M. canis* via morphological observation and internal transcribed spacer (ITS) region sequencing, with all isolates showing ≥98% sequence similarity to reference *M. canis* sequences in the NCBI Nucleotide database. WGS generated a total of 141 Gb of raw data, with de novo assembly yielding genome sizes ranging from 22.8 to 23.5 Mbp and BUSCO completeness values all exceeding 98%. Average nucleotide identity (ANI) analysis confirmed that all 38 isolates shared >99.9% sequence identity with the *M. canis* reference strain ATCC 4439 (GCF_000151145.1). Detailed clinical information (host species, age, sex) and basic genomic characteristics (contig number, total length, gene count) of the 38 isolates are summarized in Table 2.

**Figure 2:**
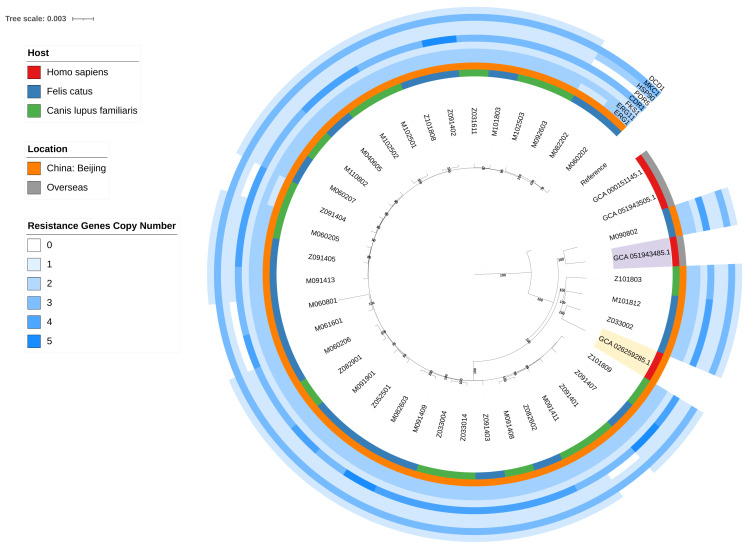
Maximum likelihood (ML) phylogenetic tree and drug resistance gene copy number distribution of 38 clinical *Microsporum canis* isolates. The phylogenetic tree was constructed based on cgSNPs, with the best-fit evolutionary model automatically selected by ModelFinder and 1000 ultrafast bootstrap tests performed to evaluate branch reliability. Tree scale bar represents 0.003 nucleotide substitutions per site. Metadata including host source (*Homo sapiens*, *Felis catus*, *Canis lupus familiaris*) and geographic origin (China: Beijing, Overseas) are annotated on the tree; the reference strain ATCC 4439 (GCF_000151145.1) was used as the outgroup. The heatmap on the right shows the copy numbers of core drug resistance genes in each isolate.

Phylogenetic analysis based on core single-nucleotide polymorphisms (cgSNPs) revealed the strong clonal homogeneity of the local *M. canis* population (Figure 2). The human-derived *M. canis* reference strain isolated from Beijing (GenBank accession: GCA_026259285.1) was fully nested within the epidemic cluster formed by local animal-derived isolates, indicating close genetic relatedness and supporting zoonotic cross-host transmission. Even a geographically distant Dutch reference strain (GCA_051943485.1) clustered closely with one local isolate, suggesting the remarkable genomic evolutionary stability of *M. canis*.

### 3.2. In Vitro Antifungal Susceptibility Phenotypes

**Figure 3:**
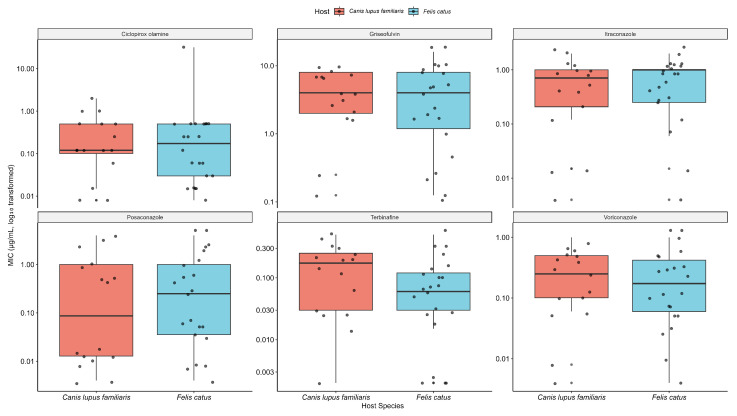
Box plots of minimum inhibitory concentration (MIC) distributions of six antifungal drugs against *Microsporum canis* isolates from *Canis lupus familiaris* and *Felis catus* hosts. Y-axis represents $\log_{10}$-transformed MIC values (μg/mL) of each antifungal drug. Box plot elements: the horizontal line inside the box indicates the median; the box limits represent the 25th (Q_1_) and 75th (Q_3_) quartiles; the whiskers extend to the minimum and maximum values within 1.5× interquartile range (IQR); individual dots represent outliers beyond 1.5× IQR. *Canis lupus familiaris*-derived isolates are marked in red and *Felis catus*-derived isolates are marked in blue (consistent with the phylogenetic tree color scheme in this study).

In vitro antifungal susceptibility testing against six clinically common antifungal drugs was performed for all 38 *M. canis* isolates (16 canine-derived, 22 feline-derived) in strict accordance with the CLSI M38 (third edition) standard. Detailed MIC values for each isolate against all six agents are provided in Table 3. Terbinafine exhibited the most potent antifungal activity, with an MIC range of 0.002 to 0.5 μg/mL (median 0.06 μg/mL). Griseofulvin (MIC range 0.125 to 16 μg/mL) and ciclopirox olamine (MIC range 0.008 to >16 μg/mL) showed weak overall antifungal activity (Figure 3).

**Figure 4:**
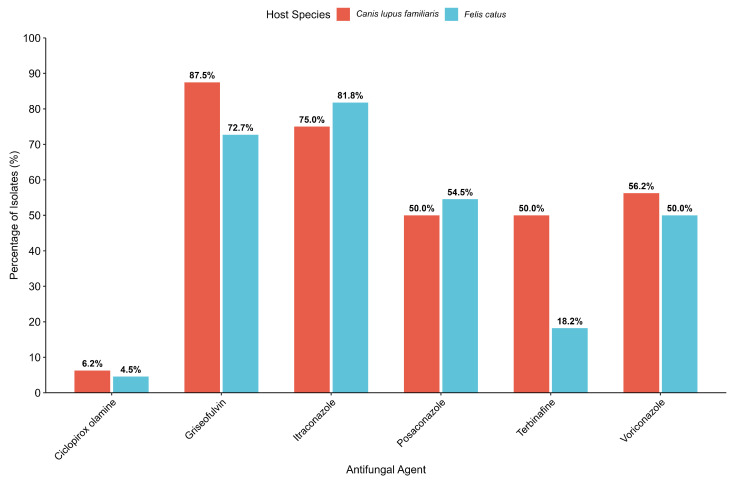
Proportion of isolates with high-MIC (non-wild type, NWT) phenotype of *Microsporum canis* between *Canis lupus familiaris*-derived and *Felis catus*-derived isolates. Y-axis represents the percentage of isolates with high-MIC phenotype for each tested antifungal drug. High-MIC thresholds were defined according to the upper limit of wild-type (UL-WT) criteria set in the Methods section: terbinafine ≥ 0.125 μg/mL, itraconazole ≥ 0.25 μg/mL, voriconazole ≥ 0.125 μg/mL, posaconazole ≥ 0.25 μg/mL, ciclopirox olamine ≥ 2 μg/mL, griseofulvin ≥2 μg/mL. Statistical analysis: Two-sided Fisher’s exact test was used to compare the difference in high-MIC proportion between the two host groups; no statistically significant difference was observed for all tested drugs (all p>0.05).

Stratified analysis by host source showed that canine-derived isolates had a numerically higher proportion of high-MIC (non-wild type, NWT) phenotype across all six tested drugs. In particular, the high-MIC rate of terbinafine in canine-derived isolates (50.0%, 8/16) was numerically higher than that in feline-derived isolates (18.2%, 4/22). Two-sided Fisher’s exact test showed no statistically significant difference in the proportion of high-MIC isolates between the two host groups for any tested agent (all p>0.05, Figure 4). A total of 11 isolates (28.9% of all tested isolates) exhibited a multidrug high-MIC phenotype.

### 3.3. Genomic Distribution of Virulence Factors and Resistance Genes

To assess whether the numerical difference in high-MIC phenotype proportion between canine-derived and feline-derived isolates was driven by genomic variations, we first analyzed the distribution of core resistance-related genes by host source. Wilcoxon rank-sum test showed no statistically significant difference in the total cumulative copy number of detected core resistance genes between the two host groups (W=142, p=0.34, Figure 7A). There was also no significant difference in the detection rate and copy number distribution of individual core resistance genes (including *CDR1*, *PDR5*, *ERG11*, *ERG1*) between canine and feline isolates (all p>0.05). Clustering analysis based on resistance gene copy number profiles showed no distinct host-specific clustering pattern, with isolates from both hosts randomly distributed across clusters.

**Figure 5:**
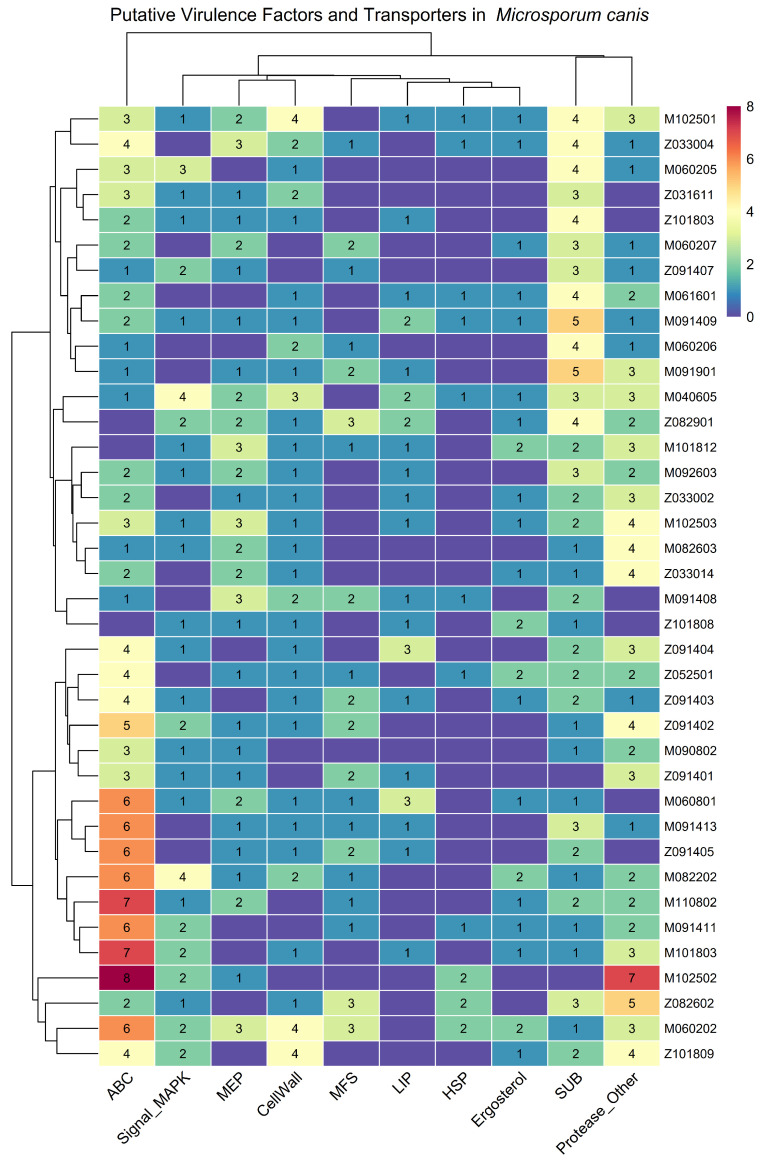
Distribution of copy numbers of virulence factor and transporter protein genes in 38 *Microsporum canis* isolates. The heatmap shows the copy number of each target gene in individual isolates. Color scale represents gene copy number, ranging from 0 (white) to 10 (dark red). Core gene families include secreted proteases (SUB, MEP, DPP), ABC transporters, MFS transporters, and cell wall biosynthesis-related genes. Note: A copy number of 0 for cell wall-related genes indicates incomplete identification of homologous genes during genome prediction and annotation, not the actual absence of cell wall structures in the isolates.

Genomic prediction of virulence factors showed that the composition of core virulence gene families was highly conserved across all isolates, with no host-specific gene deletion or copy number variation (CNV) observed. Secreted protease families associated with keratin degradation and host invasion showed significant gene enrichment: the subtilisin (SUB) family maintained three to eight copies in all strains, while the dipeptidyl peptidase (DPP) and metalloprotease (MEP) families also showed consistent copy number distribution. The ABC transporter family and major facilitator superfamily (MFS) transporters were also significantly enriched, with MFS transporters maintaining four to five copies in all isolates (Figure 5).

**Figure 6:**
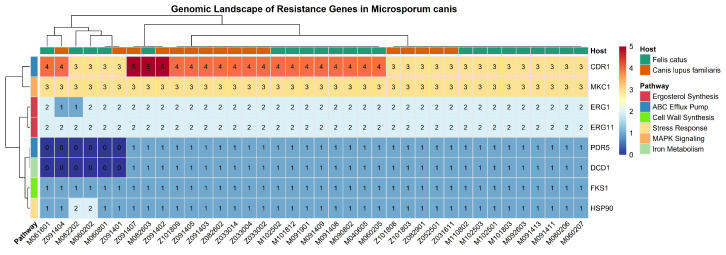
Genomic landscape of copy numbers of core drug resistance-related genes in 38 *Microsporum canis* isolates. The heatmap shows the copy number of each drug resistance-related gene in individual isolates, with host source annotated on the top. Color scale represents gene copy number, ranging from 0 (white) to 5 (dark red). The functional pathway of each gene is annotated on the left, including ergosterol biosynthesis, ABC efflux pump, cell wall synthesis, stress response, MAPK signaling, and iron metabolism.

Homologous alignment identified core antifungal resistance-related genes including ergosterol biosynthesis genes (*ERG1*, *ERG11*), ABC efflux pumps (*CDR1*, *PDR5*), cell wall synthesis gene (*FKS1*), stress response gene (*HSP90*), MAPK signaling gene (*MKC1*) and iron metabolism gene (*DCD1*). Among these, only the ABC transporter-encoding gene *CDR1* showed significant CNV among different strains, ranging from three to five copies, while the copy numbers of other resistance-related genes were relatively stable (Figure 6).

**Figure 7:**
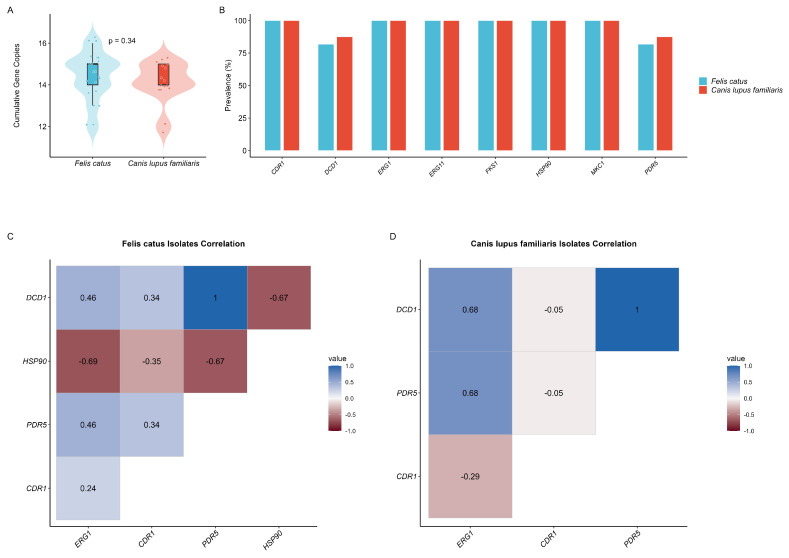
Cumulative copy numbers of core drug resistance genes and gene–gene correlation analysis in *Microsporum canis* isolates from canine and feline hosts. (**A**) Box plot of cumulative copy numbers of core drug resistance genes in *Felis catus* and *Canis lupus familiaris* isolates. Y-axis represents total cumulative copy number of detected resistance genes. Statistical analysis: Wilcoxon rank-sum test, W=142, p=0.34. Box plot elements are consistent with Figure 3. (**B**) Prevalence of core drug resistance genes in *Felis catus* and *Canis lupus familiaris* isolates. Y-axis represents the percentage of isolates carrying at least one copy of the corresponding gene. (**C**) Spearman rank correlation heatmap of core drug resistance genes in *Felis catus* isolates. (**D**) Spearman rank correlation heatmap of core drug resistance genes in *Canis lupus familiaris* isolates. Color scale for (**C**,**D**) represents Spearman correlation coefficient, ranging from −1 (dark blue, negative correlation) to 1 (dark red, positive correlation).

Spearman rank correlation analysis further revealed that *CDR1* copy number had no significant correlation with other core resistance-related genes (including *PDR5*, *ERG1*, *HSP90*, *DCD1*) in both canine-derived and feline-derived isolate groups (all |r|≤0.68, all adjusted p>0.05, Figure 7C,D), indicating that *CDR1* CNV is an independent genomic event in this study population, not linked to variations in other classical resistance-related genes.

### 3.4. Association Between CDR1 Copy Number Expansion and Multidrug High-MIC Phenotype

Spearman rank correlation analysis with false discovery rate (FDR) correction was performed to assess the association between core resistance gene copy numbers and log_2_-transformed MIC values of the six tested antifungal drugs. The results showed that *CDR1* copy number was significantly positively correlated with the MIC values of all six tested antifungal drugs. The strongest correlation was observed for voriconazole (Spearman r=0.60, adjusted p<0.001), followed by posaconazole (r=0.59, adjusted p<0.001), terbinafine (r=0.57, adjusted p<0.001), itraconazole (r=0.52, adjusted p=0.0009), and ciclopirox olamine (r=0.45, adjusted p=0.0045). No significant correlation was observed between *CDR1* copy number and griseofulvin MIC (r=0.30, adjusted p=0.065).

**Figure 8:**
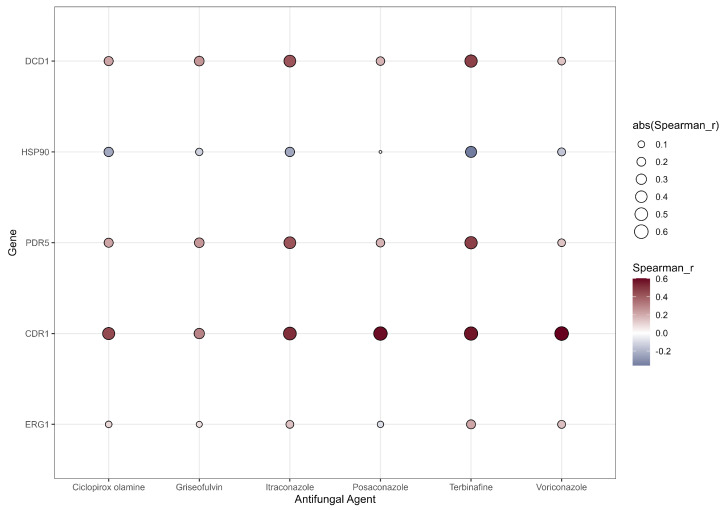
Spearman rank correlation analysis between resistance gene copy number and $\log_{2}$-transformed antifungal MIC values. Circle size represents the magnitude of the Spearman correlation coefficient (r), with larger circles indicating stronger correlation. Color scale represents the Spearman correlation coefficient, ranging from −1 (dark blue, negative correlation) to 1 (dark red, positive correlation). Statistical analysis: Spearman rank correlation with FDR correction for multiple testing. Core significant results: *CDR1* copy number was significantly positively correlated with voriconazole (r=0.60, adjusted p<0.001, $R^{2}=0.339$), posaconazole (r=0.59, adjusted p<0.001, $R^{2}=0.356$), terbinafine (r=0.57, adjusted p<0.001, $R^{2}=0.319$), itraconazole (r=0.52, adjusted p=0.0009, $R^{2}=0.200$), and ciclopirox olamine (r=0.45, adjusted p=0.0045, $R^{2}=0.194$); *PDR5* copy number was significantly positively correlated with terbinafine (r=0.47, adjusted p=0.0028, $R^{2}=0.267$) and itraconazole (r=0.42, adjusted p=0.0083, $R^{2}=0.066$).

Univariate linear regression further showed that *CDR1* copy number explained 35.6% of the variance in posaconazole MIC ($R^{2}=0.356$), 33.9% of the variance in voriconazole MIC ($R^{2}=0.339$), 31.9% of the variance in terbinafine MIC ($R^{2}=0.319$), 20.0% of the variance in itraconazole MIC ($R^{2}=0.200$), and 19.4% of the variance in ciclopirox olamine MIC ($R^{2}=0.194$). *PDR5* copy number also showed a moderate positive correlation with the MIC values of terbinafine (r=0.47, adjusted p=0.0028) and itraconazole (r=0.42, adjusted p=0.0083). No significant correlation was observed between CNV of *ERG1*, *HSP90*, *DCD1* and the MIC values of all tested drugs (all adjusted p>0.05, Figure 8).

**Figure 9:**
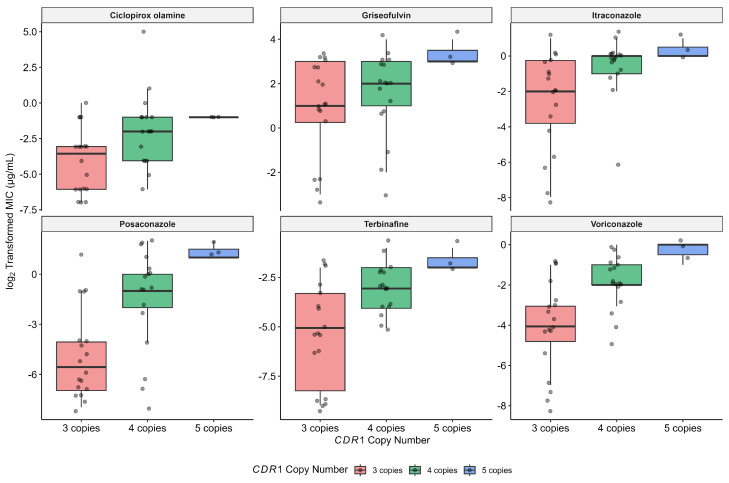
Association between *CDR1* copy number and $\log_{2}$-transformed antifungal MIC values in 38 *Microsporum canis* isolates. Y-axis represents $\log_{2}$-transformed MIC values (μg/mL) of each tested antifungal drug; X-axis represents *CDR1* copy number (3, 4, 5 copies). Box plot elements are consistent with Figure 3. Statistical analysis: Kruskal–Wallis nonparametric test was used to compare the differences in MIC values among groups with different *CDR1* copy numbers; detailed statistics: voriconazole $\chi^{2}=13.89$, df=2, p<0.001; posaconazole $\chi^{2}=13.06$, df=2, p=0.0015; terbinafine $\chi^{2}=12.37$, df=2, p=0.0021; itraconazole $\chi^{2}=9.93$, df=2, p=0.0070; ciclopirox olamine $\chi^{2}=7.58$, df=2, p=0.0226; griseofulvin $\chi^{2}=5.41$, df=2, p=0.0669.

Kruskal–Wallis nonparametric test confirmed that the MIC values of voriconazole ($\chi^{2}=13.89$, df=2, p<0.001), posaconazole ($\chi^{2}=13.06$, df=2, p=0.0015), terbinafine ($\chi^{2}=12.37$, df=2, p=0.0021), itraconazole ($\chi^{2}=9.93$, df=2, p=0.0070), and ciclopirox olamine ($\chi^{2}=7.58$, df=2, p=0.0226) increased significantly with elevated *CDR1* copy number (Figure 9). No significant association was observed between *CDR1* copy number and griseofulvin MIC ($\chi^{2}=5.41$, df=2, p=0.0669, $R^{2}=0.085$).

## 4. Discussion

In this study, we performed integrated genomic and phenotypic characterization of 38 clinical *Microsporum canis* isolates from companion animals in Beijing, China. Our core findings are fourfold: (1) *M. canis* strains exhibit high clonal homogeneity, without significant host-specific genetic differentiation between canine and feline isolates; (2) a local human *M. canis* strain is fully nested within the clade of pet isolates, providing direct genome-level phylogenetic evidence for zoonotic cross-host transmission; (3) terbinafine shows the most potent in vitro antifungal activity against local epidemic strains, while 28.9% of isolates exhibit a multidrug high-MIC phenotype; (4) copy number expansion of the ABC transporter-encoding gene *CDR1* is the key genomic feature associated with elevated MICs to multiple antifungal agents in *M. canis*. These findings fill critical gaps in the current understanding of *M. canis* zoonotic transmission and antifungal resistance, and provide a scientific basis for clinical precision therapy and One Health-based zoonotic disease control.

From a zoonotic transmission perspective, the high clonal homogeneity of *M. canis* strains, combined with the highly conserved distribution of core virulence gene families, indicates that this fungus has no observable host adaptation barrier during transmission between canine and feline hosts. Notably, the local human-derived *M. canis* strain was fully nested within the animal-derived epidemic clade, providing direct genome-level phylogenetic evidence that companion animal-derived strains can be transmitted to humans via close contact, and confirming pets as the primary reservoir hosts for human *M. canis* infection. Currently, global antifungal resistance surveillance for dermatophytes is largely focused on human clinical strains, with limited systematic monitoring of companion animal-derived epidemic strains [8,13]. Our data strongly support the integration of companion animal dermatophyte surveillance into public health frameworks to establish a human–animal integrated monitoring network and curb the cross-host transmission of drug-resistant *M. canis*.

Our in vitro antifungal susceptibility data provide clear guidance for clinical empirical treatment of *M. canis*. Terbinafine exhibited the strongest antifungal activity against all tested isolates, with the lowest MIC range (0.002–0.5 μg/mL) and median value (0.06 μg/mL), which is consistent with the 27-year large-scale epidemiological data of *M. canis* in mainland China. This finding supports terbinafine as the preferred first-line empirical treatment option for companion animal *M. canis*-associated dermatophytosis in this region. In contrast, griseofulvin and ciclopirox olamine showed weak overall antifungal activity, with individual isolates exhibiting extremely high MIC values exceeding the wild-type threshold, suggesting that these agents should be used with caution in clinical settings without prior susceptibility testing. Stratified analysis by host source showed that canine-derived isolates had a numerically higher proportion of high-MIC phenotype across all six tested agents, although the difference was not statistically significant. This observation is consistent with previous regional studies reporting similar host-associated differences in antifungal susceptibility profiles of companion animal-derived dermatophytes [43]. This observation still has clinical relevance, reminding clinicians to be alert to the risk of empirical azole treatment failure for canine-derived *M. canis* infections.

Importantly, 28.9% of isolates in this study met the definition of multidrug high-MIC phenotype, indicating that multidrug reduced susceptibility is not uncommon in local *M. canis* epidemic strains. This rate is significantly higher than the 15.2% reported in a 2025 nationwide study of 348 *M. canis* isolates from mainland China [13], suggesting a potentially higher prevalence of efflux-mediated cross-resistance in the Beijing region, which aligns with the global alarming trend of rising antifungal resistance in dermatophytes [44]. The significant positive correlation between *CDR1* copy number and MICs of antifungals with distinct mechanisms of action (allylamines, triazoles, and hydroxypyridones) provides direct evidence for cross-resistance mediated by broad-spectrum efflux pumps in *M. canis*. This cross-resistance pattern is consistent with previous reports in other dermatophytes such as *Trichophyton rubrum*, where overexpression of ABC transporters confers reduced susceptibility to multiple drug classes [45,46].

While our study focused on MIC-based resistance phenotypes, it is important to distinguish between resistance, tolerance, and heteroresistance. Tolerance refers to the ability of fungi to survive high drug concentrations without growth, while heteroresistance describes the presence of subpopulations with reduced susceptibility within a clonal isolate. Although we did not perform specific tolerance or heteroresistance assays, the wide range of MIC values observed for individual drugs (e.g., terbinafine MIC range 0.002–0.5 μg/mL) and the presence of isolates with intermediate MICs may indicate the existence of heteroresistant subpopulations. This hypothesis is supported by a recent scoping review that identified emerging evidence of heteroresistance to terbinafine in zoonotic dermatophytes [2], highlighting the need for further investigation into these understudied phenotypes in *M. canis*.

We found that copy number expansion of the ABC transporter-encoding gene *CDR1* is the key genomic feature associated with elevated MICs to multiple antifungal agents in *M. canis*, and this copy number variation is an independent genomic event not linked to variations in other classical resistance-related genes. This finding aligns with established antifungal resistance mechanisms in pathogenic fungi: *CDR1* encodes a broad-spectrum ABC efflux pump that can mediate the efflux of antifungal agents with distinct mechanisms of action, whereas variations in ergosterol biosynthesis target genes only affect susceptibility to a single drug class [25,45,46]. Under the selective pressure of widespread clinical use of multiple antifungal agents, copy number variation in broad-spectrum efflux pumps confers a greater survival advantage than single-target gene mutations, a pattern widely documented in *Candida albicans* and *Aspergillus fumigatus* [45,47]. The gene dosage effect of *CDR1* copy number expansion explained 20–36% of the variance in MIC values for five antifungal agents except griseofulvin, indicating that increased copy number directly enhances the efflux capacity of the ABC transporter. No significant association was observed between *CDR1* copy number and griseofulvin MIC ($R^{2}=0.085$, p=0.0669). We therefore propose a mechanistic model for multidrug reduced susceptibility in *M. canis*: *CDR1* copy number expansion increases the expression dosage of the broad-spectrum ABC efflux pump, enhances the efflux of multiple classes of antifungal drugs, and ultimately leads to elevated MIC values and multidrug high-MIC phenotype. This model links genomic variation to phenotypic changes in *M. canis* drug resistance and provides a clear framework for subsequent functional validation. Notably, this is the first systematic study to demonstrate the association between *CDR1* copy number variation and multidrug reduced susceptibility in *M. canis*, complementing a 2025 case report that identified *CDR1* overexpression in a single terbinafine-resistant isolate [18].

Several limitations of this study should be noted. First, we only confirmed the association between *CDR1* copy number expansion and elevated MIC phenotype at the genomic level, with no transcriptomic validation of *CDR1* expression or functional verification via gene manipulation assays, leaving the complete causal relationship not fully elucidated. Second, all isolates were collected from two veterinary hospitals in Beijing with a limited sample size, so the generalizability of our findings to other geographic regions requires further validation in multi-center, large-sample epidemiological studies. Third, this study focused only on known classical antifungal resistance-related genes, without genome-wide variant screening, which may miss other novel genomic variations associated with reduced antifungal susceptibility in *M. canis*. We did not perform a genome-wide association study (GWAS) due to the extremely high clonal homogeneity of our study population (average nucleotide identity > 99.9%), which would result in insufficient statistical power to detect significant associations between genetic variants and phenotypes, as previously demonstrated in clonal microbial populations [19,31].

Regarding the choice of antifungal susceptibility testing method, we adopted the CLSI M38 (third edition) standard rather than the EUCAST method for two main reasons. First, the CLSI M38 (third edition) provides validated quality control ranges for all six antifungal agents tested in this study (terbinafine, itraconazole, voriconazole, posaconazole, griseofulvin, and ciclopirox olamine) using *Trichophyton mentagrophytes* ATCC MYA-4439 as the quality control strain. In contrast, the current EUCAST E.Def 9.3.2 standard does not include quality control ranges for griseofulvin and ciclopirox olamine for dermatophytes, and the quality control range for terbinafine is not yet fully validated for *M. canis* [48]. Second, the vast majority of previous antifungal susceptibility studies of *M. canis* in China have used the CLSI method [13], allowing for direct comparison of our results with existing epidemiological data. Notably, fluconazole was not included in this study because neither CLSI nor EUCAST provides validated quality control ranges for fluconazole against *M. canis*, which would compromise the reliability of susceptibility testing results. This is consistent with previous observations that fluconazole exhibits poor activity against *M. canis* and is not recommended for clinical use [11,13].

## 5. Conclusions

This study provides a comprehensive genomic and phenotypic characterization of clinical *M. canis* isolates circulating in Beijing, China. We demonstrate the high clonal homogeneity of the local *M. canis* population and provide genome-level evidence for zoonotic cross-host transmission between companion animals and humans. Terbinafine exhibits the most potent in vitro antifungal activity against local epidemic strains, while multidrug high-MIC is prevalent in nearly 30% of isolates. Copy number expansion of the ABC transporter-encoding gene *CDR1* is significantly associated with elevated MICs to multiple antifungal agents, representing a key genomic marker of multidrug reduced susceptibility in *M. canis*. These findings fill critical gaps in the understanding of *M. canis* zoonotic transmission and antifungal resistance, and provide a scientific basis for clinical precision therapy, antifungal resistance surveillance, and One Health-based zoonotic disease control.

## Figures and Tables

**Table 1:** Quality control metrics of whole-genome sequencing data for 38 *Microsporum canis* isolates.

Isolate ID	Raw Reads (Pairs)	Clean Reads (Pairs)	Clean Data (Gb)	Q20 (%)	Q30 (%)	GC Content (%)	Average Sequencing Depth (×)	Genome Accession Number
M040605	22,730,982	22,690,198	3.40	99.39	97.35	47.44	151.37	JBXXSW000000000
M060202	22,907,932	22,762,050	3.41	99.40	97.40	47.75	151.82	JBXXSX000000000
M060205	24,001,608	23,933,300	3.59	99.19	96.30	47.39	159.83	JBXXSY000000000
M060206	23,279,912	23,229,574	3.48	99.47	97.52	47.42	154.94	JBXXSZ000000000
M060207	25,606,896	25,409,974	3.81	99.48	97.75	47.41	169.63	JBXXTA000000000
M060801	25,951,640	25,789,172	3.87	99.37	97.01	47.49	172.30	JBXXTB000000000
M061601	22,311,638	22,202,698	3.33	99.51	97.58	47.38	148.26	JBXXTC000000000
M082202	21,153,192	20,336,276	3.05	99.37	97.35	47.48	135.79	JBXXTD000000000
M082603	25,807,616	25,722,530	3.86	99.30	96.59	47.44	171.85	JBXXTE000000000
M090802	25,395,752	25,346,490	3.80	99.39	97.06	47.45	169.18	JBXXTF000000000
M091408	28,587,790	28,519,732	4.28	99.40	97.20	47.43	190.55	JBXXTG000000000
M091409	28,167,288	28,102,310	4.22	99.42	97.20	47.42	187.88	JBXXTH000000000
M091411	23,594,246	23,472,062	3.52	99.45	97.56	47.60	156.72	JBXXTI000000000
M091413	28,122,536	28,061,558	4.21	99.43	97.33	47.41	187.44	JBXXTJ000000000
M091901	26,951,354	26,892,934	4.03	99.43	97.29	47.41	179.42	JBXXTK000000000
M092603	20,965,316	20,855,326	3.13	99.51	97.54	47.41	139.35	JBXXTL000000000
M101803	20,989,196	20,920,266	3.14	99.39	96.97	47.48	139.80	JBXXTM000000000
M101812	21,138,104	21,027,810	3.15	99.35	96.99	47.40	140.24	JBXXTN000000000
M102501	27,948,542	27,833,332	4.17	99.43	97.78	47.45	185.66	JBXXTO000000000
M102502	26,205,946	26,093,134	3.91	99.47	97.83	47.56	174.08	JBXXTP000000000
M102503	23,407,404	23,300,372	3.50	99.45	97.61	47.44	155.83	JBXXTQ000000000
M110802	19,595,956	19,488,038	2.92	99.36	97.29	47.71	130.00	JBXXTR000000000
Z031611	21,363,662	20,961,530	3.14	99.46	97.56	47.43	139.80	JBXXTS000000000
Z033002	24,605,166	24,542,894	3.68	99.46	97.66	47.46	163.84	JBXXTT000000000
Z033004	24,577,940	24,430,164	3.66	99.55	97.73	47.39	162.95	JBXXTU000000000
Z033014	23,267,582	22,882,380	3.43	99.49	97.66	47.46	152.71	JBXXTV000000000
Z052501	24,691,794	24,616,992	3.69	99.50	97.81	47.41	164.29	JBXXTW000000000
Z082602	25,302,840	25,220,574	3.78	99.45	97.70	47.51	168.29	JBXXTX000000000
Z082901	22,783,052	22,706,882	3.41	99.39	97.07	47.61	151.82	JBXXTY000000000
Z091401	27,783,520	27,727,830	4.16	99.40	97.16	47.46	185.21	JBXXTZ000000000
Z091402	27,480,178	27,410,536	4.11	99.43	97.30	47.46	182.98	JBXXUA000000000
Z091403	27,288,104	27,219,128	4.08	99.42	97.29	47.40	181.65	JBXXUB000000000
Z091404	28,425,614	28,366,468	4.25	99.41	97.29	47.42	189.22	JBXXUC000000000
Z091405	31,572,980	31,503,450	4.73	99.43	97.30	47.41	210.59	JBXXUD000000000
Z091407	33,550,378	33,477,372	5.02	99.41	97.24	47.36	223.50	JBXXUE000000000
Z101803	20,675,358	20,611,610	3.09	99.40	97.09	47.48	137.57	JBXXUF000000000
Z101808	20,685,462	20,607,186	3.09	99.40	97.12	47.46	137.57	JBXXUG000000000
Z101809	21,155,098	21,089,580	3.16	99.40	97.09	47.53	140.69	JBXXUH000000000

**Table 2:** Clinical information and basic genomic characteristics of 38 *Microsporum canis* clinical isolates.

Isolate ID	Host Species	Breed	Age ^1^	Sex	Source Hospital	Isolate Month	Contigs	Total Length (Mb)	Genes	tRNA	ANI (%) ^2^	Genome Accession Number
M040605	*Felis catus*	American Shorthair	2 y	Female	MZAH ^3^	April	207	23.08	8252	94	99.95	JBXXSW000000000
M060202	*Felis catus*	Devon Rex	2 y	Male	MZAH	June	508	23.50	15,633	156	99.95	JBXXSX000000000
M060205	*Canis lupus familiaris*	Golden Retriever	3 y	Male	MZAH	June	185	23.14	8163	92	99.94	JBXXSY000000000
M060206	*Felis catus*	British Shorthair	3 y	Male	MZAH	June	186	23.11	8835	101	99.94	JBXXSZ000000000
M060207	*Felis catus*	Maine Coon	3 y	Female	MZAH	June	194	23.11	8347	96	99.95	JBXXTA000000000
M060801	*Felis catus*	Devon Rex	2 m	Female	MZAH	June	267	23.02	12,012	153	99.95	JBXXTB000000000
M061601	*Felis catus*	British Longhair	4 y	Male	MZAH	June	170	23.14	8325	91	99.95	JBXXTC000000000
M082202	*Felis catus*	British Shorthair	9 m	Male	MZAH	August	212	23.05	14,081	149	99.95	JBXXTD000000000
M082603	*Felis catus*	Golden British Shorthair	4 y	Female	MZAH	August	213	23.10	8203	92	99.95	JBXXTE000000000
M090802	*Felis catus*	Devon Rex	10 m	Male	MZAH	September	198	23.18	8522	97	99.95	JBXXTF000000000
M091408	*Canis lupus familiaris*	Toy Poodle	8 y	Male	MZAH	September	190	23.10	8417	109	99.95	JBXXTG000000000
M091409	*Felis catus*	British Shorthair	5 y	Female	MZAH	September	202	23.11	8377	97	99.95	JBXXTH000000000
M091411	*Canis lupus familiaris*	Samoyed	6 y	Male	MZAH	September	364	23.32	11,153	112	99.94	JBXXTI000000000
M091413	*Felis catus*	British Shorthair	3 y	Male	MZAH	September	181	23.12	8680	102	99.95	JBXXTJ000000000
M091901	*Felis catus*	British Shorthair	8 m	Female	MZAH	September	193	23.12	8440	100	99.95	JBXXTK000000000
M092603	*Canis lupus familiaris*	Yorkshire Terrier	3 y	Male	MZAH	September	173	23.11	8581	96	99.95	JBXXTL000000000
M101803	*Felis catus*	Devon Rex	3 y	Female	MZAH	October	209	23.06	8458	93	99.95	JBXXTM000000000
M101812	*Felis catus*	Devon Rex	3 m	Male	MZAH	October	219	23.17	8324	94	99.95	JBXXTN000000000
M102501	*Canis lupus familiaris*	Maltese	1 y	Female	MZAH	October	258	23.13	10,588	113	99.95	JBXXTO000000000
M102502	*Canis lupus familiaris*	Golden Retriever	2 y	Female	MZAH	October	313	23.18	10,389	112	99.95	JBXXTP000000000
M102503	*Canis lupus familiaris*	Bichon Frise	1 y	Male	MZAH	October	221	23.13	9809	104	99.95	JBXXTQ000000000
M110802	*Canis lupus familiaris*	Schnauzer	10 y	Male	MZAH	November	490	23.63	11,533	119	99.94	JBXXTR000000000
Z031611	*Canis lupus familiaris*	Welsh Corgi	4 y	Male	CAUVTH ^4^	March	213	23.09	8294	97	99.95	JBXXTS000000000
Z033002	*Felis catus*	British Shorthair	9 m	Male	CAUVTH	March	165	23.12	8266	97	99.95	JBXXTT000000000
Z033004	*Canis lupus familiaris*	Toy Poodle	11 y	Female	CAUVTH	March	160	23.14	8382	96	99.95	JBXXTU000000000
Z033014	*Canis lupus familiaris*	Shiba Inu	2 y	Female	CAUVTH	March	215	23.08	8191	92	99.94	JBXXTV000000000
Z052501	*Felis catus*	Munchkin	4 m	Female	CAUVTH	May	188	23.12	8432	94	99.94	JBXXTW000000000
Z082602	*Felis catus*	Domestic Shorthair	2 m	Male	CAUVTH	August	215	23.02	8393	99	99.95	JBXXTX000000000
Z082901	*Canis lupus familiaris*	Maltipoo	6 m	Male	CAUVTH	August	188	22.90	8737	98	99.96	JBXXTY000000000
Z091401	*Canis lupus familiaris*	French Bulldog	4 y	Male	CAUVTH	September	210	23.04	8721	99	99.95	JBXXTZ000000000
Z091402	*Felis catus*	Ragdoll	3 y	Female	CAUVTH	September	207	23.08	8462	101	99.95	JBXXUA000000000
Z091403	*Felis catus*	Domestic Shorthair	4 m	Female	CAUVTH	September	175	23.27	8526	104	99.95	JBXXUB000000000
Z091404	*Canis lupus familiaris*	Toy Poodle	10 y	Male	CAUVTH	September	187	23.10	8445	102	99.95	JBXXUC000000000
Z091405	*Felis catus*	Selkirk Rex	1 y	Male	CAUVTH	September	177	23.19	8536	103	99.95	JBXXUD000000000
Z091407	*Felis catus*	Chinese Li Hua	1 y	Male	CAUVTH	September	220	23.26	8662	126	99.95	JBXXUE000000000
Z101803	*Canis lupus familiaris*	Schnauzer	12 y	Male	CAUVTH	October	229	23.15	8610	99	99.95	JBXXUF000000000
Z101808	*Felis catus*	British Shorthair	3 y	Female	CAUVTH	October	199	23.08	8485	100	99.95	JBXXUG000000000
Z101809	*Canis lupus familiaris*	Bichon Frise	1 y	Male	CAUVTH	October	275	23.10	8464	103	99.96	JBXXUH000000000

^1^ Age is presented as year (y) for adult animals and month (m) for juvenile animals; 1 y = 12 months. ^2^ ANI: Average Nucleotide Identity, calculated against the reference strain ATCC 4439 (GCF_000151145.1). ^3^ Meilian Zhonghe Animal Hospital. ^4^ China Agricultural University Veterinary Teaching Hospital.

**Table 3:** Minimum inhibitory concentrations (MICs) of six antifungal drugs against 38 clinical *Microsporum canis* isolates (unit: μg/mL).

Isolate ID	Voriconazole	Terbinafine	Itraconazole	Ciclopirox Olamine	Posaconazole	Griseofulvin
M040605	0.25	0.12	1	0.06	0.25	16
M060202	0.12	0.002	0.12	0.03	0.03	1
M060205	0.5	0.25	0.5	0.12	4	4
M060206	0.12	0.06	0.5	0.5	0.5	2
M060207	0.06	0.015	0.06	0.015	0.03	2
M060801	0.5	0.002	0.25	0.12	0.06	8
M061601	0.25	0.03	0.015	0.03	0.004	0.5
M082202	0.06	0.002	0.25	0.015	0.5	4
M082603	1	0.25	2	0.5	2	8
M090802	0.25	0.06	1	0.25	1	8
M091408	0.5	0.12	1	0.5	0.5	4
M091409	0.25	0.06	0.25	>16	0.5	8
M091411	0.12	0.03	0.25	0.008	0.5	2
M091413	0.03	0.03	1	0.015	0.008	0.25
M091901	0.25	0.12	2	0.5	2	4
M092603	0.06	0.015	0.015	0.12	0.004	8
M101803	0.008	0.06	0.5	0.5	0.06	4
M101812	0.06	0.12	1	0.06	0.06	0.125
M102501	0.25	0.12	2	0.5	2	8
M102502	0.25	0.25	1	0.5	4	4
M102503	0.5	0.25	1	1	0.015	8
M110802	0.004	0.002	0.004	0.008	0.008	0.25
Z031611	0.5	0.25	1	0.015	0.008	2
Z033002	1	0.25	1	0.5	4	8
Z033004	0.5	0.5	1	1	1	8
Z033014	1	0.25	1	0.06	0.015	2
Z052501	0.06	0.12	1	0.06	0.06	2
Z082602	0.03	0.03	1	0.015	0.008	0.25
Z082901	0.06	0.25	0.015	0.12	0.004	8
Z091401	0.008	0.03	0.12	0.12	0.015	0.125
Z091402	1	0.25	1	0.5	4	8
Z091403	0.12	0.06	1	0.25	0.25	2
Z091404	0.12	0.06	0.5	0.25	0.5	2
Z091405	0.5	0.12	0.5	0.25	1	4
Z091407	0.5	0.5	1	0.5	2	16
Z101803	0.12	0.03	0.5	0.12	0.015	8
Z101808	0.004	0.002	0.004	0.008	0.008	0.125
Z101809	0.5	0.5	2	2	1	8

## Data Availability

The de novo assembled whole-genome sequences of the 38 *Microsporum canis* isolates generated in this study have been deposited in the NCBI GenBank database. All related data are publicly accessible under the BioProject accession number **PRJNA1460975**, and the corresponding GenBank genome accession number for each isolate is detailed in Table 1.

## Supplementary Materials

The following supporting information can be downloaded at https://www.mdpi.com/article/10.3390/jof12060429/s1. Table S1: Information of antifungal drug-related genes in *Microsporum canis* and reference fungal species.

## Abbreviations

The following abbreviations are used in this manuscript:

ANI	Average Nucleotide Identity
ABC	ATP-binding Cassette
CLSI	Clinical and Laboratory Standards Institute
CNV	Copy Number Variation
DMSO	Dimethyl Sulfoxide
FDR	False Discovery Rate
ITS	Internal Transcribed Spacer
IQR	Interquartile Range
MFS	Major Facilitator Superfamily
MIC	Minimum Inhibitory Concentration
ML	Maximum Likelihood
NWT	Non-Wild-type
PCR	Polymerase Chain Reaction
RPMI	Roswell Park Memorial Institute
SDA	Sabouraud Dextrose Agar
SNP	Single Nucleotide Polymorphism
UL-WT	Upper Limit of Wild-type
WGS	Whole-genome Sequencing
